# Perceptions of Stress and Engagement in High-Intensity Caregiving: A Cross-Sectional Study in Lithuania

**DOI:** 10.1177/00469580241290081

**Published:** 2024-11-05

**Authors:** Lina Jaruseviciene, Apolinaras Zaborskis, Aurelija Blazeviciene, Leonas Valius, Ausrine Kontrimiene

**Affiliations:** 1 Lithuanian University of Health Sciences (LUHS), Kaunas, Lithuania

**Keywords:** caregiver, informal care, home nursing, long-term care, mental health, caregiving stress, primary health care, caregiving engagement, high-intensity caregiving

## Abstract

The aging population and overstretched healthcare systems are increasing demand for home nursing by informal caregivers, significantly affecting their mental health. This study aimed to examine the level of population’s engagement in caregiving and the association between high-intensity caregiving and perceived caregiving stress in the general population of Lithuania. A nationally representative sample (N = 1000) of Lithuanian residents aged 18 years and older (mean age 53.1 ± 17.9 years) was interviewed in their households. The results showed that 17.4% (95% CI: 15.1, 19.8) of respondents were involved in home nursing activities to some extent, with 42% of these being high-intensity caregivers (providing 11 or more hours of home care per week). Caregivers were statistically significantly more likely to be female and have higher education levels. Perceived stress was significantly associated with higher education levels (OR = 2.66, 95% CI: 1.41, 5.02), high-intensity caregiving (OR = 2.14, 95% CI: 1.15, 3.97), regular involvement in home nursing (OR = 1.86, 95% CI: 1.01, 3.43), and caring for recipients with dementia or individuals entirely dependent on assistance (OR = 2.52, 95% CI: 1.22, 5.23). Caregivers perceived stress is associated with their level of education, the intensity and regularity of home nursing, as well as the level of dependency of the care recipient, especially in cases of dementia. Comprehensive long-term care policies should be developed to ensure the larger availability of formal care resources, increased societal participation in home nursing, and tailored interventions for high intensity caregivers.

**What do we already know about this topic?**
Increased level of home nursing workload is adversely associated with health - predicts higher caregiver strain and challenge their mental health. In countries with fewer formal long-term care resources and a reliance on traditional caregiving practices, the proportion of intensive caregiving is typically higher. The Central and Eastern Europe region has been identified as having the most harmful effects on the mental health of informal caregivers.
**How does your research contribute to the field?**
The research makes a clear distinction between high-intensity and low-intensity caregiving, highlighting the importance of recognizing the diverse challenges faced by different caregivers for a more nuanced understanding. Recognizing this diversity is essential for crafting targeted interventions tailored to the unique needs of various caregiver groups, which could include routine psychological assessments for those providing high-intensity care. The study, drawing upon data from Lithuania—a country in Central and Eastern Europe (CEE)—enhances our comprehension of how cultural and structural determinants distinctively influence the caregiving experience across various nations.
**What are your research’s implications toward theory, practice, or policy?**
The study reveals that higher educational attainment predicts caregiver stress, challenging traditional views that associate higher stress with lower education. This suggests a need to revise theoretical models to consider stressors for educated caregivers. Furthermore, suggests the need of tailored interventions and routine psychological assessments for high-intensity caregivers.

## Introduction

A growing elderly population is leading to a greater demand for caregiving services, particularly due to disability, illness, or frailty.^1,2^ As healthcare systems become increasingly overstretched,^3^ there is a growing trend toward the privatization and marketization of long-term care (LTC),^4^ which particularly burdens socioeconomically disadvantaged households.^5^ In response, informal caregiving, typically provided by family or friends, takes on the responsibility of delivering extensive home nursing and support.^6^

Increased level of home nursing workload is adversely associated with health - predicts higher caregiver strain^7-9^ with women at greater risk of distress^10,11^ and challenge their mental health.^12^ Research indicates that the increased risk of depressive symptoms and mortality is especially pronounced among in-home caregivers, such as spouses or adult children living with the care recipient.^13^ This could be attributed to the fact that in-home caregivers often feel a more significant duty to fulfill caregiving responsibilities compared to out-of-home caregivers, who may view caregiving as more voluntary.^8^ Additionally, greater involvement in caregiving correlates with lower education and lower income,^14^ which can impact both health and labor market participation.^2^

Research indicate that higher proportion of intensive caregivers is characteristic of countries with fewer formal LTC resources.^14,15^ Comparatively low state responsibility for LTC and reliance on traditional modes of caregiving are typical in the Central and Eastern European (CEE) region. This region also displays deep fragmentation between health and social sectors,^16^ strong family care norms, and less developed social protection networks.^17^ Studies addressing the context specific nature of caregivers wellbeing in Europe distinguish Southern & Eastern Cluster (including CEE countries) as the regions with the most harmful effects on mental health for informal caregivers^18^ and stress the need for major policy efforts in these regions.^17^

Lithuania could serve as a representative example of LTC in the CEE region. LTC expenditure in Lithuania is 1.1% of GDP, compared to the OECD average of 1.5%, and there is only 1 formal LTC worker per 100 people aged 65 or above, compared to the EU average of 4.1. According to Eurostat, professional home care services in Lithuania are provided to less than 9% of households in need, which is less than half of the EU average.^19^ Consequently, LTC in Lithuania predominantly relies on informal caregivers. Approximately 90% of Lithuanians receive exclusively informal caregiving, significantly exceeding the EU average (70%), with caregivers providing care intensively.^16^

Economic forecasts suggest that the public sector will face considerable strain аs the share of the population aged 65 years and over is expected to grow from 20% in 2019% to 32% in 2050.^16^ Faster aging rate compared to the OECD and EU averages, complemented by factors such as youth emigration, high female employment, and an increase in single-person households further indicate the high unsustainability of relying on informal care.^20^

Qualitative surveys performed in Lithuania indicated the high burden of informal caregiving, affecting also psychological wellbeing of caregivers and pointed out the lack of systemic measures to improve the situation.^21,22^ Lithuanian survey of non-random sample of informal caregivers revealed that 77.4% of participants were attributed to the group in intensive caregiving and confirmed high intensity home nursing among predictors of caregivers burden.^19^ Meanwhile data from European Social Survey performed in 2014 to 2015 revealed Lithuania as the country with lowest population involvement in caregiving—20.4% comparing to 34.24%—mean of 19 European countries, with the intensive caregiving reaching the average—6.8%.^14^

Given the increasing demand for informal caregiving, our study aimed to examine populations’ engagement in caregiving and the relationship between high-intensity caregiving and perceived stress. The survey took place in September 2020, coinciding with a period of relative tranquility before the onset of the second wave of COVID-19, following the initial outbreak.

## Materials and Methods

### Study Design and Participants

This study was a part of a larger project titled “Integrated Health Care for Senior Mental Health: Developing an Intersectoral Cooperative Care Model,” funded by the Lithuanian Research Council (Project ID: S-MIP-17-121) and spanning 3 years (2017-2020). The project’s goal was to assess the healthcare and social care needs of older adults with dementia and their family caregivers, aiming to improve services through intersectoral collaboration.^22-24^

The present paper focuses specifically on the findings from a survey conducted among the Lithuanian general population in September 2020. This study adopted a cross-sectional design. Sample size calculation was performed with G*Power (Version 3.1.9.4, University of Düsseldorf, Düsseldorf, Germany) program, based on a two-tail *z*-test. A minimum sample of n = 776 participants was calculated to detect differences between any 2 independent proportions, given 95% confidence interval (CI), power 80% and a medium effect size of 10%. The result was doubled because it was expected that only 50% of the sample could participate in the study. Thus, at least 1552 people should be recruited into the current study.

A random multilevel sampling procedure was employed to draw a nationally representative sample. Initially, 27 towns and 42 villages of Lithuania were selected, then 1786 residents aged 18 and older were selected from them. The sample drawn ensured representativeness of the country population by age, sex, education, employment, and size of residential location. The sampling of the participants and the subsequent their survey were conducted by “Vilmorus Ltd, Lithuania,” an agency for public opinion and market research (http://www.vilmorus.lt/en).

For inclusion in the study, participants needed to meet the following criteria: they had to be 18 years or older and reside in Lithuania. Additionally, eligibility required participants to be available at home during the survey period. Lastly, participants were required to voluntarily consent to participate after being fully informed about the study’s purpose, the selection procedures, and the assurances of confidentiality that were in place.

The respondents were interviewed in their household. However, 358 residents were not at home during the survey period, and an additional 428 residents declined to participate. If the intended resident was not available, they could be replaced by a spouse or a parent, resulting in 74 (7.4%) respondents being substituted in this way. As a result, 1000 participants completed the survey, representing a response rate of 56%. The respondents were interviewed in their household. Prior to oral interview, study participants were provided with written information detailing the selection procedure, purpose of the survey, and intended publications. They were assured of full confidentiality.

We followed recommendations for Observational Studies in Epidemiology (cross-sectional studies) and the use of the STROBE Checklist in reporting results (see Supplemental Materials).

### Questionnaire and Study Variables

A questionnaire (see Supplemental Materials), specifically designed for this study, was used to conduct interviews. It included 32 questions, covering respondents’ socio-demographic information and various aspects of the home nursing experience (home nursing refers to the medical care administered in a residential setting, where individuals can receive personalized assistance from family members or friends).

Prior to conducting the main survey, the questions underwent a pilot test with a sample size of ten participants (n = 10) to ensure their comprehensibility. This study analyzes the following variables.

The background variables (covariates) included respondents’ socio-demographic information: gender (male or female), age, education, familial status, personal income, and place of residence. Considering the retirement age limit established in the Republic of Lithuania, age was dichotomized into 2 categories: <65 years and ≥65 years. Education was categorized into higher (college or university) and less than higher (secondary, basic, or primary) education. Familial status was grouped into 2 categories: married/partnership and living without a partner (unmarried, divorced, widowed). Personal income was assessed as ≤400 EUR/month and >400 EUR/month, using the median value as a cutoff point. Place of residence was classified as urban or rural based on the selected towns or villages.

Respondents were classified as informal caregivers if they responded “yes, regularly” or “yes, occasionally” to the question “Do you personally provide home nursing to a family member or close friend?.” Those informal caregivers who dedicated 11 or more hours per week to caregiving were further categorized as high intensity caregivers.^15,25^

The outcome variable for the study was caregiving stress perception. The exposure to stress was measured using the following single question: “Do you feel stressed when you think about your personal experience providing home nursing to another person?” For analysis, the respondents were divided into those who felt stressed (response options: “I feel very stressed”/“I feel stressed”/“I feel a moderate stress”/“I feel a little stress”) and those who did not feel stressed (response options: “I don’t feel stressed”/“I don’t think about stress”). The question was asked only to those respondents who were classified as informal caregivers. A similar single-item stress measure has been shown to have satisfactory content, criterion, and construct validity for survey research.^26^

In addition to socio-demographic variables, a set of predictors was included in this study to predict caregiving stress perception. Respondents were asked if they provide home nursing and to whom, categorized by social relationship (eg, family member, neighbor, friend) or by age and degree of dependence on others (eg, elderly person 65 years or older who is totally dependent on help). Additionally, we inquired about who assists the respondents in providing home nursing, allowing for multiple-choice answers.

### Data Analysis

Data analysis was conducted using the Statistical Package for the Social Sciences (SPSS) for Windows (version 21.0; SPSS Inc, Chicago, IL, USA, 2012). Continuous numerical variables were described as mean ± standard deviation (SD). Categorical variables were presented as absolute number of cases (n) and percentages (%). Contingency tables with the Chi-square (χ^2^) test were employed to explore statistical relationship between categorical variables. The binary logistic regression analysis was used to identify predictors of caregiver’s stress perception and need of emotional support by estimating odds ratio (OR) with its 95% confidence interval (CI). The adjusted odds ratios (aOR) quantified the strength of association between each predictor and psycho-emotional factor after accounting for potential socio-demographic confounders. For all analyses, the level of statistical significance was set at P < .05.

### Research Ethics

The study was granted approval in 2018 (Approval No. BE-2-47) by the Kaunas Regional Biomedical Research Ethics Committee, ensuring compliance with ethical standards. Respondents were thoroughly informed regarding the objectives of the study and were explicitly told that they had the full right to withdraw their participation at any moment. Furthermore, verbal consent was secured from every participant before proceeding with the study.

## Results

### Sample Characteristics

Table 1 shows socio-demographic characteristics of respondents. Of the 1000 respondents, 53.0% were females. The mean age of the respondents was 53.1 ± 17.9 years, so 29.3% of respondents were 65 years old and older; 58.1% reported being married or living in cohabitation, 67.6% were from urban area. Almost equal proportions of respondents had a higher (college or university) education and less than higher education (47.6% and 52.4%, respectively), the median of income per capita was 400 EUR/month.

**Table 1.** Socio-demographic Characteristics of Respondents.

Characteristics	Total		Caregivers		Non-caregivers	
	n	(%)	n	(%)	n	(%)
Gender						
Males	470	(47.0)	67	(38.5)	403	(48.8)
Females	530	(53.0)	107	(61.5)	423	(51.2)
			χ^2^(1, N = 1000) = 6.10; *P* = .014			
Age						
Mean (SD)	53.1	(17.9)	54.7	(14.9)	52.7	(18.4)
			*t(998)* = 1.35; *P* = .176			
<65 years	707	(70.7)	130	(74.7)	577	(69.9)
≥65 years	293	(29.3)	44	(25.3)	249	(30.1)
			χ^2^(1, N = 1000) = 1.64; *P* = .201			
Education						
Secondary or less	524	(52.4)	77	(44.3)	447	(54.1)
College or university	476	(47.6)	97	(55.7)	379	(45.9)
			χ^2^(1, N = 1000) = 5.61; *P* = .018			
Familial status						
Married	481	(48.1)	95	(54.6)	386	(46.7)
Cohabitation	100	(10.0)	20	(11.5)	80	(9.7)
Unmarried	134	(13.4)	14	(8.0)	120	(14.5)
Divorced	129	(12.9)	25	(14.4)	104	(12.6)
Widower	156	(15.6)	20	(11.5)	136	(16.5)
			χ^2^(4, N = 1000) = 9.45; *P* = .051			
Residence						
Urban area	676	(67.6)	117	(67.2)	559	(67.7)
Rural area	324	(32.4)	57	(32.8)	267	(32.3)
			χ^2^(1, N = 1000) = 0.01; *P* = .911			
Personal income						
≤400 EUR/month	436	(43.6)	75	(49.7)	361	(49.9)
>400 EUR/month	438	(43.8))	76	(50.3)	362	(50.1)
Missing value	126	(12.6)	χ^2^(1, N = 874) = 0.01; *P* = .953			

From the total sample, 17.4% (95% CI: 15.1, 19.8) reported involvement to some extent in home nursing activities (caregivers). Compared to non-caregivers, caregivers were statistically more likely to be older, female, have a college or university education, and, to some extent, to be a married person. Place of residence (urban/rural) and personal income did not have a statistically significant relationship with involvement in caregiving (Table 1).

### Intensity of Caregiving and Care Recipients’ Profiles

Table 2 presents respondents’ responses regarding home nursing and the recipients of such care, comparing the frequency of reports between groups categorized by low and high intensity caregiving. As can be seen, there were 101 (58.0%) and 73 (42.0%) caregivers in the respective groups.

**Table 2.** Overview of Home Nursing Engagement, Care Recipients, and Caregiving Intensity Among Respondents.

Home nursing engagement	Total		Level of caregiving intensity			
			Low (<11 h per week of caregiving)		High (≥11 h per week of caregiving)	
	n	(%)	n	(%)	n	(%)
Involved in home nursing	174	(17.4^a^)	101	(58.0)	73	(42.0)
Average time spent on home nursing (±SD), hours/week	26.17 ± 41.54		5.33 ± 2.97		56.51 ± 51.84	
Care recipients by social relationship:						
Family member who lives together	64	(36.0)	13	(20.3)	51	(79.7)
Family member who lives separately	78	(43.8)	63	(80.8)	15	(19.2)
Neighbor	23	(12.9)	17	(73.9)	6	(26.1)
Friend who lives together	11	(6.2)	11	(100)	0	(0)
Beneficiary of volunteer organization	2	(1.1)	1	(50.0)	1	(50.0)
Total	178^b^	(100)	105	(59.0)	73	(41.0)
			χ^2^(4, N = 178) = 62.19; *P* < .001^d^			
Care recipients by age and level of dependence on assistance:						
Person with senile dementia	15	(8.3)	5	(33.3)	10	(66.7)
65 years or older person who partially depend on the help of others	117	(64.5)	76	(65.9)	41	(34.1)
65 years or older person who entirely depend on the help of others	24	(13.3)	8	(33.3)	16	(66.7)
Adult under the age of 65 years	20	(11.1)	15	(75.0)	5	(25.0)
Child under the age of 18 years	5	(2.8)	2	(40.0)	3	(60.0)
Total	181^c^	(100)	106	(58.6)	75	(41.4)
			χ^2^(4, N = 181) = 15.14; *P* = .004^d^			
Regularity of involvement in home nursing						
Regularly	74	(42.5)	27	(36.5)	47	(63.5)
Occasionally	100	(57.5)	74	(74.0)	26	(26.0)
Total	174	(100)	101	(58.0)	73	(42.0)
			χ^2^(1, N = 174) = 24.58; *P* < .001^d^			

a Percents from the total sample.

b One respondent reported 3 subjects, 3 respondents reported 2 persons to whom they are providing home nursing, and 1 respondent didn’t report to this item.

c One respondent reported 3 persons, 8 respondents reported 2 persons to whom they are providing home nursing, and 3 respondents didn’t report to this item.

d Significance of the difference between the answers of respondents with low and high involvement in home nursing.

Respondents in the high intensity caregiving group spent, on average, ten times more hours in caregiving compared to those in the low intensity group (56.51 ± 51.84 vs 5.33 ± 2.97). Furthermore, they were more likely to care regularly than occasionally (63.5% vs 36.5%) and primarily provided care for family members living together (79.7% of cases), whereas respondents in the low intensity caregiving group more often provided home nursing for family members living separately, neighbors, or friends.

Statistically significant differences were observed between the high and low intensity caregiving in terms of the care recipient profile. Specifically, respondents in the high intensity group reported more frequently caring for individuals with senile dementia (60% vs 40%), older individuals who were entirely dependent on the assistance of others (66.7% vs 33.3%), and individuals younger than 18 years (60% vs 40%).

There were no statistically significant differences between low and high-intensity caregiving with respect to socio-demographic factors or the assistance received in the provision of home nursing services. Approximately half (48.9%) of the caregivers reported that they are solely responsible for home nursing and do not receive assistance.

### Caregiving Stress Perception

Among the respondents participating in home nursing, 72 (41.4%) reported feeling stressed and 31 (17.8%) reported need for emotional support when thinking about their personal experience of caring.

Multivariable logistic regression was used to analyze factors associated with the perception of caregiving stress (Table 3). The individual factors significantly associated with higher odds of perceived stress included college or university education (OR = 2.66, 95% CI: 1.41, 5.02, *P* < .01), high intensity home nursing (OR = 2.14, 95% CI: 1.15, 3.97, *P* < .05), regular involvement in home nursing (OR = 1.86, 95% CI: 1.01, 3.43, *P* < .05), and caring for a recipient with dementia or an older individual entirely dependent on the assistance (OR = 2.52, 95% CI: 1.22, 5.23, *P* < .05). The strength of association of perceived caregiving stress with the characteristics of caregivers did not change noticeably when the data were adjusted for socio-demographic variables. Therefore, it can be concluded that perceived stress is significantly associated with high-intensity caregiving, regular involvement in home nursing, and caring for recipients with dementia or individuals entirely dependent on assistance. Higher education was a significant confounder when adjusting all these predictors for socio-demographic variables.

**Table 3.** Factors Associated With Perception of Caregiving Stress.

Predictors	Total, N	Cases, n (%)	OR (95% CI)	aOR (95% CI)
Gender				
Males	67	28 (41.8)	1	
Females	107	44 (41.1)	0.97 (0.52, 1.81)	
Age				
<65 years	130	56 (43.1)	1	
≥65 years	44	16 (36.1)	0.76 (0.37, 1.53)	
Education				
Secondary or less	77	22 (28.6)	1	
College or university	97	50 (51.5)	2.66 (1.41, 5.02)**	
Marital status				
Married or partnership	115	46 (40.0)	1	
Other status	59	26 (44.1)	1.18 (0.63, 2.23)	
Residence				
Urban area	117	50 (42.7)	1	
Rural area	57	22 (38.6)	0.84 (0.44, 1.61)	
Personal income				
≤400 EUR/month	75	31 (41.3)	1	
>400 EUR/month	76	36 (47.4)	1.28 (0.67, 2.43)	
Intensity of involvement in home nursing				
Low	101	34 (33.7)	1	1
High	73	38 (52.1)	2.14 (1.15, 3.97)*	2.83 (1.44, 5.57)**
Regularity of involvement in home nursing				
Occasionally	100	35 (35.0)	1	1
Regularly	74	37 (50.0)	1.86 (1.01, 3.43)*	2.06 (1.08, 3.93)*
Does anyone assist you in providing home nursing?				
No	85	32 (37.6)	1	1
Yes	89	40 (44.9)	1.35 (0.74, 2.48)	1.36 (0.70, 2.65)
Caring for recipients with dementia or >65-year olds entirely dependent on the assistance				
No	135	49 (36.3)	1	1
Yes	39	23 (59.0)	2.52 (1.22, 5.23)*	2.72 (1.27, 5.86)**

*Note.* OR = crude odds ratio; AOR = odds ratio adjusted for gender, age, education, marital status, and residence (personal income was not used for this purpose due to 23 missing its values).

* *P* < .05. **P < .01.

## Discussion

The findings of the study revealed that a significant proportion of participants were involved in long-term home nursing with nearly half of them dedicating over 11 h weekly to high-intensity caregiving tasks. Furthermore, caregivers were statistically significantly more likely to be female, older, and have higher education levels. While other studies also show similar trends in gender and age,^27-29^ the educational level of caregivers varies. Some studies report a higher proportion of caregivers with lower education levels^30^ while others, including those in Lithuania, find a predominance of caregivers with education beyond high school.^24,31,32^ This inconsistency might be due to selection bias and differences in the general population’s educational level in each country. Lithuania ranks among the top 5 OECD countries for educational attainment among individuals aged 25 to 64.^23^ However, the larger proportion of caregivers with higher education in our study may be related to other factors, such as greater health literacy, access to resources, flexible job conditions, and financial stability, which enable individuals with higher education to provide care more effectively.^31,33,34^ These aspects merit further research to understand the higher representation of educated individuals among informal caregivers.

Higher education in our study was also a significant predictor of perceived caregiving stress. This correlation is uncommon. For instance, a systematic analysis by Maximiano-Barreto et al^35^ identified low schooling as a factor associated with a higher caregiving burden. However, our findings align with some previous research. A Canadian study by Chappell et al^36^ indicated a higher caregiving burden among those with higher education when caring for persons with dementia. Similarly, a national survey in the USA found that higher education increases the physical aspect of the caregiving burden^30^ and a population survey in Germany concluded that higher education increases the odds of feeling mentally burdened.^31^ Explanations suggest that higher-educated caregivers may fear a loss of self-fulfillment and autonomy^31^ or prefer to spend their time on other activities.^33,36^ Further investigation, particularly through qualitative studies, is needed to better understand the unique stressors faced by highly educated caregivers. It is possible that caregivers with lower education levels might perceive home nursing as a more natural part of life than highly educated caregivers—understanding these correlations could be instrumental in developing tailored interventions for caregivers.

Our study reveals distinct profiles among informal caregivers. The majority of caregivers spend an average of 5 h per week (5.33 ± 2.97 h) on home nursing, typically supporting family members living separately, neighbors, or friends. In contrast, a significant group, constituting 42% of all caregivers, spends over 55 h per week on caregiving, usually for cohabitating family members with serious conditions like dementia or complete dependency. High-intensity caregivers experience higher levels of stress, this finding is consistent with other studies.^24,37,38^

Non homogeneity of informal caregivers’ profiles suggests the need for diversified support strategies. Horizontal approaches are needed to increase social participation in caregiving. Our study found that nearly half of Lithuanian caregivers are solely responsible for home nursing, possibly due to prevailing social norms and constitutional mandates for children to care for their parents.^4^ Research indicate that larger familiarity with caregiving, appreciation of informal caregivers, deeper cooperation between informal and professional caregivers might be instrumental in reducing public stigma toward informal caregiving.^39,40^ These community-driven solutions complemented with caregivers’ training could be helpful in expanding caregivers’ ranks, in improving quality of care and in reducing caregiver stress.^21,41^

Additionally, a vertical approach, as a targeted, specialized strategy designed to address the specific needs of high-intensity caregivers is essential. Firstly, supplementing existing formal home care services with routine assessments of caregivers could be instrumental in detecting alarming symptoms and needs. Providing home nursing for 11 or more hours per week^10,24^ could serve as a red flag for routine psychological assessments and potential referrals to mental health services. Secondly, our findings suggest that comprehensive long-term care management algorithms might be helpful, particularly when starting with conditions such as dementia and those involving complete dependency on assistance. Studies indicate that comprehensive dementia care management not only improves care for individuals with dementia but also significantly reduces caregivers’ feelings of being overwhelmed.^11^ Our study serves as a valuable stepping stone for deeper investigation, employing more robust measures to determine the potentially optimal points of intervention. Thirdly, the expansion of formal long-term care infrastructure in Lithuania warrants consideration as a significant step toward improving the nation’s healthcare services. More generous long-term care infrastructure, as shown by Verbakel et al,^14^ is associated with a lower proportion of high-intensity caregivers.

This study has several limitations. First, our survey was conducted in September 2020, between the first and second waves of COVID-19. Although quarantine restrictions, including the temporary suspension of formal home care services, had been lifted by the time of our survey, these prior restrictions, and other experiences during the first COVID-19 wave may have significantly influenced the general perception of stress and caregiving stress in particular. Studies in other countries show inconsistent findings regarding the prevalence and intensity of informal care during the pandemic—some indicate that informal care remained relatively unchanged,^42^ while others report increased efforts by informal caregivers.^42^ There is still a common agreement that the psychological well-being of caregivers was adversely affected.^42^

Second, the study was cross-sectional, and therefore cannot prove a causal relationship. Third, the variables used in this study were rather limited and did not fully reflect the wide spectrum of factors related to the intensity of caregiving. While we included hours per week allocated for caregiving, we did not account for the length of caregiving, nursing during the night, sleep duration, etc.

Regarding the validation of the questionnaire, we wish to clarify that the nature and scope of our study warranted a bespoke approach. Hence, we developed a specialized questionnaire tailored specifically to our research objectives. Given this customization, traditional validation was not pursued: most of the questions were intended to clarify different parts of the home nursing process, so they did not form groups of questions, commonly called scales. Therefore, in this case, no scale validation tests were applied (eg, Cronbach’s alpha was not calculated). Instead, we opted for a comprehensive pilot-testing phase to ensure the reliability and relevance of our questionnaire to the target population. Furthermore, caregiving stress was measured by a single question. However, our large sample population survey representing the whole country suggests that this single question may be an effective indicator for the routine assessment of the psychological well-being of informal caregivers. The literature presents studies indicating that the single stress-symptoms item demonstrates satisfactory content, criterion, and construct validity for group-level analysis, thus the longer scales used to measure psychological stress can be replaced with single-item measure in survey research.^26^ Future research on the clinical value of this approach in directing informal caregivers to appropriate resources would be beneficial.

## Conclusions

Informal caregiving in Lithuania is a significant and complex aspect of societal care, with many caregivers dedicating substantial time to high-intensity tasks, particularly for family members with severe conditions like dementia. Caregiver perceived stress is influenced not only by the intensity and regularity of caregiving but also by factors such as the caregiver’s education level and the recipient’s level of dependency. To avoid significant resource depletion in informal care, it is crucial to achieve a more equitable balance of the caregiving burden between family members and the state. The findings highlight the need for diversified support strategies, including broad community-based initiatives to increase social engagement in caregiving and targeted interventions for high-intensity caregivers. Expanding formal long-term care infrastructure and implementing routine psychological assessments for caregivers could be instrumental in alleviating their burden and enhancing their well-being.

## Supplemental Material

sj-docx-1-inq-10.1177_00469580241290081 – Supplemental material for Perceptions of Stress and Engagement in High-Intensity Caregiving: A Cross-Sectional Study in Lithuania Supplemental material, sj-docx-1-inq-10.1177_00469580241290081 for Perceptions of Stress and Engagement in High-Intensity Caregiving: A Cross-Sectional Study in Lithuania by Lina Jaruseviciene, Apolinaras Zaborskis, Aurelija Blazeviciene, Leonas Valius and Ausrine Kontrimiene in INQUIRY: The Journal of Health Care Organization, Provision, and Financing

sj-docx-2-inq-10.1177_00469580241290081 – Supplemental material for Perceptions of Stress and Engagement in High-Intensity Caregiving: A Cross-Sectional Study in Lithuania Supplemental material, sj-docx-2-inq-10.1177_00469580241290081 for Perceptions of Stress and Engagement in High-Intensity Caregiving: A Cross-Sectional Study in Lithuania by Lina Jaruseviciene, Apolinaras Zaborskis, Aurelija Blazeviciene, Leonas Valius and Ausrine Kontrimiene in INQUIRY: The Journal of Health Care Organization, Provision, and Financing
