# Supplementary material for: Perceptions of Stress and Engagement in High-Intensity Caregiving: A Cross-Sectional Study in Lithuania
Source: Inquiry. 2024 Nov 5;61:00469580241290081. doi: 10.1177/00469580241290081 (PMC11536479; doi:10.1177/00469580241290081)
Supplement: sj-docx-1-inq-10.1177_00469580241290081 – Supplemental material for Perceptions of Stress and Engagement in High-Intensity Caregiving: A Cross-Sectional Study in Lithuania [file sj-docx-1-inq-10.1177_00469580241290081.docx]

**Questionnaire on the Care of Older Adults**

**(Aged 65 and Over) at Home**

*Long-term care and nursing at home involve organizing various services and providing financial support for individuals with disabilities who are unable to fully take care of themselves over long periods. Long-term care services are individualized, promote personal independence, enhance quality of life, and fulfill both medical and non-medical needs.*

*Home assistance - social and medical LONG-TERM assistance/care/nursing at home.*

1. **Do you need help at home with daily activities?**
2. Yes, I need help
3. Partially need help
4. No, I do not need help
5. I don't know, I don't think about it
6. **How likely is it that you personally will need help at home in the next 5 years?**
7. Very likely
8. Likely
9. Moderately likely
10. Unlikely
11. Very unlikely
12. **How likely is it that an aging family member or close friend will need help at home in the next 5 years?**
13. Very likely
14. Likely
15. Moderately likely
16. Unlikely
17. Very unlikely
18. **Do you personally provide help at home to a family member or close friend?**
19. Yes, I regularly provide such help/care
20. Yes, occasionally
21. No, I do not provide care

ASK THOSE WHO PROVIDE HELP AT HOME TO OTHERS (see Q4 = 1 or 2)

1. **Whom do you provide home care for?**
2. A family member I live with
3. A family member living separately
4. A neighbor
5. A friend I live with
6. A friend living separately
7. I participate as a volunteer in a non-governmental organization
8. Other____________________
9. **If you provide help at home, to whom?** (Multiple choice)
10. I help an elderly person suffering from senile dementia/Alzheimer's
11. I care for an elderly person (65 years and older) who is partially dependent on others' help
12. I care for an elderly person (65 years and older) who is fully dependent on others' help
13. I care for an adult (younger than 65 years) who is partially dependent on others' help
14. I care for an adult (younger than 65 years) who is fully dependent on others' help
15. I care for a child under 18 years old who is partially dependent on others' help.
16. I care for a child under 18 years old who is fully dependent on others' help.
17. No, I do not provide such care

ASK THOSE WHO THEMSELVES NEED HELP AT HOME (see Q1=1 or 2). OTHERS => skip to Q10.

1. **If you need help at home, who provides it? Mark all applicable answers.**
2. Family members
3. Friends
4. Neighbors
5. Health care professionals (e.g., doctor, nurse)
6. Social workers
7. Non-governmental organizations (e.g., Caritas, the Maltese or others)
8. Church, religious community
9. Community center
10. The local municipality or city government
11. Other (specify):_______________
12. I need this help, but I do not receive it

ASK THOSE WHO RECEIVE HELP AT HOME (see Q7 1-10).

1. **How many hours per week do you receive help at home from others (family members, friends, social workers, etc.)?**

Enter: ________________ hrs per week

1. **If you receive help, do you pay for it?**
2. Always pay
3. Sometimes pay
4. Do not pay
5. I don't know

ASK THOSE WHO PROVIDE HELP TO ANOTHER PERSON AT HOME (see Q4=1 or 2).

1. **How many hours per week do you provide help at home to another person?**

**Enter________________ hrs per week**

1. **If you provide help, do you receive compensation for it?**
2. Always receive
3. Sometimes receive
4. Do not receive
5. I don't know
6. **Does anyone help you to provide care at home to another person?**
7. Yes => go to Q13.
8. No => go to Q14.
9. **How many hours PER WEEK do other individuals/institutions help you to provide care at home to another person? Answer about everyone who helps.**

|  | **Hours per week** |
| --- | --- |
| Family members |  |
| Friends |  |
| Neighbors |  |
| Healthcare professionals (e.g., doctors, nurses) |  |
| Social workers |  |
| Non-governmental organizations (e.g., Caritas, the Maltese or others) |  |
| Church, religious communities |  |
| Community center |  |
| Eldership or municipality |  |
| Other (specify) |  |

1. **How do you feel when you think about your personal experience in providing help at home to another person?**
2. I feel very stressed
3. I feel highly stressed
4. I feel moderate stress
5. I feel a little stress
6. I do not feel stressed
7. I don't know, I don't think about it
8. **Do you yourself need help, emotional support in providing help at home to another person?**
9. Yes, very much needed
10. Yes, needed
11. Neither needed nor unneeded
12. No, not needed
13. No, absolutely not needed
14. I don't know, I don't think about it
15. **From whom do you receive emotional support at home when providing help to another person? I receive emotional support from: (choose from 1 to 5 options)**
16. From family members
17. From friends
18. From neighbors
19. From healthcare professionals (e.g., doctor, nurse)
20. From social workers
21. From non-governmental organizations (e.g., Caritas, Maltese or others)
22. From the church, religious communities
23. From the community center
24. From the eldership or municipality
25. From social networks, the internet
26. Other (specify): _______________
27. **What are the most significant negative experiences you can name when you think about your personal experience in providing help at home to another person? (choose from 1 to 5 options)**
28. Restriction/lack of social activities (e.g., meetings with friends and relatives, participation in events, etc.)
29. Restriction of hobbies and interests
30. Lack of rest
31. Lack of sleep
32. Restriction/lack of personal life, time for oneself, privacy
33. Decrease in good emotional well-being
34. Restriction/lack of freedom and independence (“not being tied down”)
35. Restriction/lack of time spent with family and friends
36. Restriction on career advancement or academic pursuits
37. I had to reduce work hours/had to quit work/could no longer work
38. Lack of money, financial difficulties/reduction in finances
39. Restriction/lack of the opportunity to leave home for a longer time, to go to other places, to travel
40. Deterioration in physical health (e.g., exacerbation of chronic diseases, the onset of new illnesses)
41. Deterioration of emotional, mental health
42. I do not see/experience any negative experiences
43. I don't know
44. Other (specify): ________________________
45. **What are the most significant positive experiences you can name when you think about your personal experience in providing help at home to another person? (choose from 1 to 5 options)**
46. Repaying a moral debt to loved ones/feeling of compensating by giving attention to a close one
47. Spending more time with loved ones
48. Strengthening of bonds, relationships with the cared-for loved ones
49. Feeling of personal growth
50. Feeling of being needed
51. Opportunity for additional earnings (receiving benefits, care money)
52. Improvement of emotional, mental health
53. Learning to manage stressful situations
54. Acquisition of new skills (e.g., caregiving)
55. Emergence of a new role/activity in life
56. Increased sense of meaning and purpose in personal life
57. Passing on/continuing family caregiving traditions
58. Demonstrating caregiving within the family and teaches children to take care of their parents or loved ones when needed
59. Satisfaction in knowing that a loved one is receiving excellent care
60. I do not see/experience any positive experiences
61. I don't know
62. Other (specify): ________________________

**19. In your opinion, what are the current sources of financial assistance provided for home care to elderly persons (aged 65 and older)?**

*Assign the number 1 to the main financial source (highest rank), and number 6 to the least significant source (lowest rank). Allocate all six ranks, from 1 to 6.*

| **Currently cared for from/by:** | **Enter a number**  **from 1 to 6** |
| --- | --- |
| Personal Savings |  |
| Additional Income (stocks, real estate income, etc.) |  |
| Pension |  |
| State Health Insurance (Compulsory Health Insurance Fund) |  |
| Private Health Insurance (life, investment insurance) |  |
| State Support (social assistance) |  |
| Other (specify): ____ |  |

**20. WHO, in your opinion, SHOULD FINANCE the long-term care at home for elderly persons (aged 65 and older)?**

*Assign the number 1 to the main financial source (highest rank), and number 6 to the least significant source (lowest rank). Allocate all six ranks, from 1 to 6.*

| Should be cared for/nursed from: | **Enter a number**  **from 1 to 6** |
| --- | --- |
| Personal savings |  |
| Additional income (stocks, real estate income, etc.) |  |
| Pensions |  |
| National health insurance (Compulsory Health Insurance Fund) |  |
| Private health insurance (life, investment insurance) |  |
| State support (social assistance) |  |
| Other (specify): ________________________________ |  |

- 1. **In your opinion, whose responsibility is it to ensure that elderly persons (aged 65 or older) receive proper home care? Whose responsibility is it? Answer for each - the state, the family, the individual themselves.**

|  | Very High Responsibility | Very High Responsibility | Very High Responsibility | Very High Responsibility | Very High Responsibility |
| --- | --- | --- | --- | --- | --- |
| State responsibility | 1 | 2 | 3 | 4 | 5 |
| Family responsibility | 1 | 2 | 3 | 4 | 5 |
| Individual's responsibility | 1 | 2 | 3 | 4 | 5 |
| Other (who?)  ____________ | 1 | 2 | 3 | 4 | 5 |

- 1. **Evaluate how well the following institutions are currently fulfilling their duty in providing home care for older individuals (aged 65 and older)?**

|  | **Very good** | **Good** | **Moderate** | **Bad** | **Very bad** |
| --- | --- | --- | --- | --- | --- |
| State | 1 | 2 | 3 | 4 | 5 |
| Family | 1 | 2 | 3 | 4 | 5 |
| The individual themselves | 1 | 2 | 3 | 4 | 5 |
| Other (who?) | 1 | 2 | 3 | 4 | 5 |

- 1. **Do you think that the state should increase funding for the organization of long-term home care for older individuals (aged 65 and older)?**

1. Yes
2. No
3. Don't know
4. **How strongly would you agree that the state should increase funding for the provision of long-term home care for older individuals (aged 65 and older)? Please rate on a scale from -5 to +5.**

| I completely disagree that the state should increase funding for the care/nursing of elderly individuals at home. | –5 –4 –3 –2 –1 0 +1 +2 +3 +4 +5 | I strongly agree that the state should increase funding for the care/nursing of elderly individuals at home. |
| --- | --- | --- |

1. **In Lithuania, a certain portion of the taxes collected from individuals (6.98 percent) is allocated specifically for the organization of healthcare in the country (Compulsory Health Insurance Fund). How strongly would you agree with the introduction of an additional tax specifically dedicated to the organization of long-term care?**

| I would completely disagree with the introduction of such a tax. | –5 –4 –3 –2 –1 0 +1 +2 +3 +4 +5 | I would strongly agree with the introduction of such a tax. |
| --- | --- | --- |

1. **How, in your opinion, could the organization of home care assistance in Lithuania be improved?**

| **Financial Sources** | **Provided now.**  **Place of importance**  **from 1 to 6** | **Should be**  **provided.**  **Place of importance**  **from 1 to 6** |
| --- | --- | --- |
| Personal savings |  |  |
| Additional income (stocks, real estate income, etc.) |  |  |
| Pensions |  |  |
| State health insurance (Compulsory Health Insurance Fund) |  |  |
| Private health insurance (life, investment insurance) |  |  |
| State support (social assistance) |  |  |
| Other (please specify): ________________________________ |  |  |
